# Roles of Extracellular HSPs as Biomarkers in Immune Surveillance and Immune Evasion

**DOI:** 10.3390/ijms20184588

**Published:** 2019-09-17

**Authors:** Eman A. Taha, Kisho Ono, Takanori Eguchi

**Affiliations:** 1 Department of Dental Pharmacology, Graduate School of Medicine, Dentistry and Pharmaceutical Sciences, Okayama University, Okayama 700-8525, Japan; pj7l8pfb@s.okayama-u.ac.jp; 2 Department of Medical Bioengineering, Graduate School of Natural Science and Technology, Okayama University, Okayama 700-8530, Japan; 3 Department of Biochemistry, Ain Shams University Faculty of Science, Cairo 11566, Egypt; 4 Department of Oral and Maxillofacial Surgery, Okayama University Hospital, Okayama 700-0914, Japan; de20012@s.okayama-u.ac.jp; 5 Advanced Research Center for Oral and Craniofacial Sciences, Graduate School of Medicine, Dentistry and Pharmaceutical Sciences, Okayama University, Okayama 700-8525, Japan

**Keywords:** heat shock protein (HSP), exosome, oncosome, alarmin, immunology, immune evasion, resistance-associated secretory phenotype (RASP), immune surveillance, hypoxia, biomarker

## Abstract

Extracellular heat shock proteins (ex-HSPs) have been found in exosomes, oncosomes, membrane surfaces, as well as free HSP in cancer and various pathological conditions, also known as alarmins. Such ex-HSPs include HSP90 (α, β, Gp96, Trap1), HSP70, and large and small HSPs. Production of HSPs is coordinately induced by heat shock factor 1 (HSF1) and hypoxia-inducible factor 1 (HIF-1), while matrix metalloproteinase 3 (MMP-3) and heterochromatin protein 1 are novel inducers of HSPs. Oncosomes released by tumor cells are a major aspect of the resistance-associated secretory phenotype (RASP) by which immune evasion can be established. The concepts of RASP are: (i) releases of ex-HSP and HSP-rich oncosomes are essential in RASP, by which molecular co-transfer of HSPs with oncogenic factors to recipient cells can promote cancer progression and resistance against stresses such as hypoxia, radiation, drugs, and immune systems; (ii) RASP of tumor cells can eject anticancer drugs, targeted therapeutics, and immune checkpoint inhibitors with oncosomes; (iii) cytotoxic lipids can be also released from tumor cells as RASP. ex-HSP and membrane-surface HSP (mHSP) play immunostimulatory roles recognized by CD91+ scavenger receptor expressed by endothelial cells-1 (SREC-1)+ Toll-like receptors (TLRs)+ antigen-presenting cells, leading to antigen cross-presentation and T cell cross-priming, as well as by CD94+ natural killer cells, leading to tumor cytolysis. On the other hand, ex-HSP/CD91 signaling in cancer cells promotes cancer progression. HSPs in body fluids are potential biomarkers detectable by liquid biopsies in cancers and tissue-damaged diseases. HSP-based vaccines, inhibitors, and RNAi therapeutics are also reviewed.

## 1. Introduction

**Figure 1:**
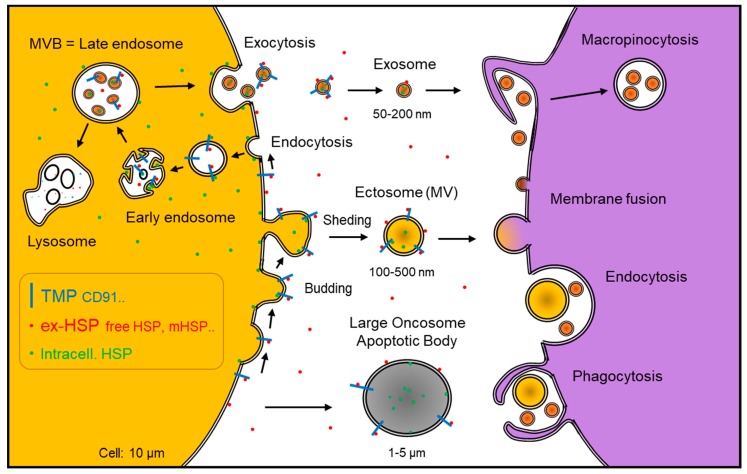
Extracellular, vesicular, and membrane heat shock proteins (HSPs). Extracellular vesicles (EVs) are often a heterogeneous mixture of exosomes, ectosomes (also known as microvesicles, MVs), oncosomes, large oncosomes, and apoptotic bodies. Exosomes are secreted via membrane fusion of multivesicular bodies (MVBs), thereby intraluminal vesicles (ILVs) are exocytosed (top left). HSP90 was shown to mediate MVB-to-plasma-membrane fusion. Distinctively, the shedding of the plasma membrane generates ectosomes (center). Cell-free, vesicle-free HSPs can be released from cells upon cell damage and stress (the so-called alarmin or chaperokine). Transmembrane proteins (TMP) such as CD91 can keep binding of HSPs on the surface of EVs and cells (blue bars and red balls). Membrane-surface HSPs (mHSP) are known as tumor antigens (red). Intracellular HSPs can be kept bound to the intracellular domains of the TMPs, such as epidermal growth factor receptor (EGFR) family members on the cells and EVs. EVs are often taken up by recipient cells in a variety of ways such as endocytosis, macropinocytosis, membrane fusion, and phagocytosis (right).

Heat shock proteins (HSP) were initially found in cells, although later studies discovered extracellularly released HSP (ex-HSP), membrane-surface HSP (mHSP), and extracellular vesicles (EVs) such as HSP-rich oncosomes/exosomes [1] (Figure 1). Tumor cells are often exposed to stresses such as hypoxic stress, immune response, inflammatory stress, microbial stimuli, and therapeutic stress. These stresses induce HSPs, molecular chaperones essential for protein folding and balancing between proteostasis and proteolysis, and play anti-apoptotic roles in cancer [2,3,4,5].

Meanwhile, ex-HSPs, including mHSP, HSP-rich exosomes, and oncosomes, play key roles in intercellular communication in cancer, immunity, and various pathological conditions (Table 1). HSPs are released from cells either by passive release, e.g., from damaged, stressed, or dead cells, or active release, including secretion of HSP-containing exosomes. Free ex-HSPs have been also called alarmin or chaperokine. Exosome HSPs were initially found in B cells, from which Hsp27, Hsc70, Hsp70, and Hsp90 are released with exosomes upon heat shock stress [6]. Proteomics revealed that oral cancer oncosomes are enriched in HSP90 members, HSP70 members, Hsc70, HSP105, and chaperonin [1]. Notably, HSP90α is highly expressed in cancer cells and secreted to extracellular space as free HSP90α [5] as well as cargo of oncosome [1]. Additionally, HSP90β, TRAP1, and some members of HSP70 are found in oncosomes [1]. However, the mechanism by which HSPs are incorporated within the vesicles and their biological significance is still unknown. We here propose two models of EV-HSPs: (i) intra-vesicular packaged HSPs and (ii) EV-membrane associated HSPs on the outer surface of EVs (Figure 1).

Ex-HSP, EV-HSP, and mHSP can bind to cell surface receptors (i) stimulating intracellular signaling pathways, (ii) taken up by endocytosis, or (iii) molecularly transferred to recipient cells (see Section 4). Such recipient cells include various immune cells, cancer cells themselves, as well as potential cancer stem cells [CSC, also known as cancer-initiating cells (CIC)], epithelial cells [7], cancer-associated fibroblasts (CAFs), mesenchymal stem cells (MSC), tumor endothelial cells (TEC), and lymphatic endothelial cells.

Small HSPs, including Hsp27 and αB-crystallin (αBC), have been detected in sera, cerebrospinal fluids, and exosomes in various pathological conditions such as cancers, neurodegeneration, and ischemia-reperfusion [8]. Extracellular small HSPs also play roles in cell–cell communication and immunomodulation. Numerous studies have reported exosomal, free, and membrane-associated HSPs (Table 1).

### 1.1. Roles of ex-HSP in Cancer Progression vs. the Host Immune System

A number of studies showed that tumor-derived exosomes promote cancer progression by transferring oncogenic factors, including oncoproteins and oncogenic miRNA (oncomiR), to recipient cells in the tumor microenvironment and in the pre-metastatic niche [25]. ex-HSP and EV-HSP play crucial roles in cancer progression by binding to cell surface receptors such as CD91 [low-density lipoprotein receptor-related protein (LRP1)/α2 macroglobulin receptor (A2MR)], promoting epithelial-mesenchymal transition (EMT), migration, invasion, heterogeneity, metastasis, CSC-like properties, and drug resistance in cancer cells and angiogenesis [26,27,28,29,30,31,32]. HSPs and oncoproteins within EVs could be a resistant-associated secretory phenotype (RASP), co-transmissive to recipient cells and leading to cancer expansion and malignant conversion of the tumor microenvironment (Figure 1). Several aspects and proof-of-concept (POC) of RASP are summarized in Section 2.

Immune cells express receptors for HSPs, such as scavenger receptor expressed by endothelial cells-1 (SREC-1), Toll-like receptors (TLRs), CD94 lectin-like natural killer receptor, and CD91/LRP1/A2MR macroglobulin receptor, leading to endocytosis, antigen cross-presentation, T cell cross-priming, and immune response. On the other hand, CD91 receptor is also expressed in cancer cells and mediates ex-HSP90 signaling, which promotes cancer progression.

ex-HSP is also found on the membrane surface of cells and EVs. ER chaperones such as binding immunoglobulin protein (BiP/HspA5) and Gp96/Grp94/Hsp90B1, known as tumor rejection antigen (TRA), can translocate to the cytosol and eventually the surface of cells during ER stress induced by drugs, UV irradiation, and microbial stimuli [33]. HSPs on the surface of cells and EVs can be recognized by CD91+ tumor cells, CD91+ fibroblasts, CD91+ SREC1+ TLR+ professional antigen presentation cells (APCs), and CD94+ cytolytic immune cells. Therefore, ex-HSP, including mHSP, can activate malignancy events in tumor cells and, in contrast, can trigger antigen cross-presentation and cross-priming by APCs and stimulate the cytolytic immune cells such as natural killer (NK) cells and CD8+ cytotoxic T lymphocytes (CTL) [34,35,36,37] (see Section 3 and Section 4).

### 1.2. HSP90 and Co-Chaperones

HSP90 homologs are the major intracellular chaperones that ensure the correct folding and function of proteins by interacting with various intracellular proteins [3,4,38,39]. HSP90 has been implicated in promoting tumor growth and metastasis of breast cancer, leukemia, pancreatic cancer, and ovarian cancer [40,41,42]. Four homologs of HSP90 are localized in different cellular compartments. HSP90α, an inducible type of HSP, and HSP90β, a constitutively expressed type of HSP, are found in the cytoplasm. Gp96/Grp94/Hsp90B1 is present in the endoplasmic reticulum (ER). Tumor necrosis factor (TNF) receptor-associated protein 1 (TRAP1) exists in the mitochondria. TRAP1 is a homolog of HSP90, although its molecular weight is 75 kD.

A number of studies have reported pathophysiological roles of HSP90 in various diseases, including bacterial and viral infection [43,44,45,46], autoimmune diseases [47,48,49,50,51], cerebrovascular diseases [52,53,54], and cancer. It is worth noting that the up-regulation of HSP90 in cancer is due to the fact that cancer cells are constantly under stressful conditions such as acidosis, hypoxia, metabolic, and nutrient deficiency [5,55,56]. High expression of HSP90 has been reported in various cancer types, including lung cancer, breast cancer, colon cancer, and blood cancer, and correlates with poor prognosis [38,57]. Intracellular HSP90 (α, β) is often called “HSP90”. HSP90 is involved in the maturation and the stabilization of a wide range of oncogenic client proteins crucial for oncogenesis and malignant progression, such as signal transduction molecules Src and RAF1, cyclin-dependent kinase-4 (CDK4), steroid hormone receptors, nitric-oxide synthase (NOS), Akt, PI3K, mutant p53 [58,59], ERBB2 (also known as HER2) [60], and HIF-1α.

A recent study showed that HSP90 mediates MVB-to-plasma-membrane fusion [61]. This study showed that HSP90 membrane-deforming ability promotes exosome release in vitro and in vivo. HSP90 possesses conserved amphipathic helix motifs that allow them to bind membranes and modulate their physical properties. Membrane deformation only occurs when HSP90 dimer is in the open state and is hindered by compounds that stabilize the closed conformation.

HSP90 has more than 10 cochaperones that cooperate with HSP90 for their clientele [39]. The stress-induced phosphoprotein 1 (Stip1) is also known as Hsp70-Hsp90 Organizing Protein (HOP) co-chaperone, inhibiting the closed HSP90 dimer conformation. Lauwers et al. showed that Stip1/HOP co-chaperone also promoted exosome release [61]. Cell division control 37 (CDC37) is a kinome co-chaperone of HSP90, regulating the folding of most kinases in the cells. CDC37 plays a crucial role as a co-chaperone of HSP90 in the stabilization of most kinases, including Src, RAF1, and CDK4, and steroid hormone receptors [62,63,64]. Therefore, HSP90 and/or CDC37 are attractive therapeutic targets against various cancers inasmuch as HSP90 and CDC37 are involved in the functionalization of oncogenic proteins in many signaling pathways important for tumor progression, survival, and resistance.

### 1.3. Inducibility of HSPs

According to their structural homologies, the HSP family members are classified into subfamilies (Table 1). From another perspective, members of the HSP family are classified into two types: an inducible type of HSPs and a constitutively expressed type of HSPs. The inducible HSPs are expressed when cells are exposed to stress such as heat and hypoxia [4,65]. Nevertheless, a number of studies have reported that both inducible and constitutive types of HSPs are often overexpressed in malignant tumors and associated with the incidence as well as the progression of the disease and the lymph node metastatic rate [56,66,67]. Consistently, inducible types of HSPs such as Hsp90α, Hsp72, Hsp70B’, and small HSPs are often found extracellularly and in EVs such as oncosomes, exosomes, and ectosomes [1,18,68,69], while constitutive HSPs such as Hsp90β and Hsc70 have been found in oncosomes [1] (Table 1).

Heat shock factor 1 (HSF1) is a canonical transcription factor that mediates cell stress and induces the production of HSPs [3]. HSF1 often collaborates with hypoxia-inducible factor 1 (HIF-1), mediating hypoxia stress response. HSP90 has more than 10 co-chaperones that define roles of the HSP90/co-chaperone complex and their clientele spectrum. Cell division control 37 (CDC37) is a kinome co-chaperone of HSP90, regulating the folding of most kinases in the cells. CDC37 induction is reciprocally regulated by two SCAN-type transcription factors—myeloid zinc-finger 1 (MZF1) and SCAN domain-containing protein 1 (SCAN-D1)—in prostate cancer [62,70].

Inducible types of HSP genes including HSP72, HSP70B’, and HSP27 are also induced by intracellular matrix metalloproteinase-3 (MMP-3) and heterochromatin protein 1 (HP1), also known as chromobox proteins (CBX) activate *HSP* genes [68].

Genetic amplification of *HSP* genes found in particular types of cancer can cause high expression of HSPs [2], while genetic mutations in *HSP* genes have barely been found, suggesting epigenetic involvement of HSPs in tumor mutation burdens (TMB).

### 1.4. Table of Contents

1. Introduction (Section 1)
2. RASP (Section 2)
3. Immunology of HSPs (Section 3)
4. Receptors for HSPs (Section 4)
5. Inducibility of HSPs and co-chaperone (Section 5)
6. HSPs as biomarkers detectable by liquid biopsies (Section 6)
7. HSP-targeted therapeutics (Section 7)
8. Conclusions (Section 8)

## 2. Resistance-Associated Secretory Phenotype (RASP)

### 2.1. HSP-Rich, Oncoprotein-Rich EVs

HSPs are often carried by EVs, e.g., exosomes, oncosomes, and microvesicles (MVs, also known as ectosomes), as EV cargos and/or are associated on the surface of EVs [1,5] (Figure 1). EV-mediated molecular transfer of oncoproteins such as mutant epidermal growth factor receptor (EGFR) and amplified HSPs [2] can enhance carcinogenesis in surrounding recipient cells such as cancer cells themselves, normal epithelial cells, fibroblasts, adipocytes, endothelial cells, macrophages, and other immune cells [1,7,71]. As EV-free HSPs do, HSPs associated with the surface of EVs could activate receptors such as CD91 and promote cancer cell EMT, migration, invasion, heterogeneity, angiogenesis, metastasis, and drug resistance. Thus, EV-HSP and ex-HSP are major aspects of the RASP.

### 2.2. Ejection of Drugs and Antibodies with HSP-EVs

The RASP is also important in drug resistance inasmuch as cancer cells are able to eject molecularly targeted drugs with EVs. Particularly, molecularly targeted anti-EGFR antibody drug Cetuximab is able to bind to EGFR and inhibit EMT, a key step in cancer progression [7]; however, oral cancer cells ejected Cetuximab with EGFR-containing EVs in response to administration of Cetuximab, indicating a novel EV-mediated mechanism of drug resistance, a POC of RASP [72]. The antibody drugs can recruit Fc receptor (FcR)-expressed immune cells, leading to phagocytosis by macrophages and/or cytolysis by CTLs and by NK cells, although these anti-cancer immune cells can be released with EVs from cancer cells. The EV-mediated ejection of drugs is a new manner of drug resistance in cancer cells as well as a novel aspect of RASP.

Anticancer drugs can cause the release of exosomes with HSPs, consistent with the concept of RASP. As another POC, anticancer drugs caused the release of exosomes with HSPs from human hepatocellular carcinoma cells, although the released HSP-exosomes elicited effective NK cell antitumor responses in vitro [73], suggesting an immunostimulatory role of EV-HSP.

### 2.3. Release of Redundant Toxic Lipids

Lipid efflux is the other aspect of RASP. Redundant lipids are released from cells through the release of lipid-layered EVs and lipid cholesterol efflux pump proteins. Such a pump overexpressed in metastatic cancer cells was adenosine triphosphate (ATP)-binding cassette G1 (ABCG1) [74]. Targeted silencing of ABCG1 resulted in the accumulation of EV lipid and triggered cell death in tumors, suggesting that cancer cells can often release redundant toxic lipid, whereas loss of the ABCG1 pump could trigger the accumulation of redundant, toxic lipids. Thus, the release of redundant, toxic EV lipids can be the other aspect of RASP, whereas the accumulation of the redundant lipid could be toxic to tumor cells, suggesting a conceptually and substantially novel therapeutic approach.

## 3. Immunomodulatory Roles of ex-HSP

Both the immunostimulatory and the immunosuppressive roles of ex-HSPs have been reported (Table 2). The immunostimulatory ex-HSPs have been reported as HSP-peptide complex vaccines to stimulate anti-tumor immunity. On the other hand, the immunosuppressive ex-HSP has been reported as microbial HSP70/HSP60 inducing dendritic cell (DC) tolerance and stimulating immunosuppressive cells such as myeloid-derived suppressor cells (MDSCs) and regulatory T cells (Tregs) in tolerating chronic inflammatory diseases such as rheumatoid arthritis (RA), type 1 diabetes, and atherosclerosis.

### 3.1. Immunogenic Immunostimulatory Roles of ex-HSP

A number of studies reported antitumor immunostimulatory roles of HSP-peptides complex vaccines. HSP-peptide complexes vaccination elicits anti-tumor immunity or other cells used as the source of HSPs, suggesting that HSP-peptide complexes can be useful against cancers and infectious diseases [75] (Table 3). From the aspect of the immune surveillance system, ex-HSPs released from damaged cells as a danger signal and/or damage-associated molecular patterns (DAMP) can stimulate professional APCs, followed by the release of cytokines and the expression of cell surface molecules [76,77,78]. Moreover, ex-HSPs can promote the cross-presentation of HSP-bound peptide antigens to major histocompatibility complex (MHC) class I molecules in DCs, efficiently inducing antigen-specific CTLs. The roles of HSPs stimulating both innate immunity and adaptive immunity may be the molecular mechanism by which thermal stress bolsters the host immune system [79]. HSP peptide complexes vaccination induces antigen cross-presentation by professional APCs such as DCs and macrophages [80], thereby eliciting HSP-cross-primed antigen-specific CD8+ CTLs [34,35,36,37]. The HSP peptides vaccines have been examined in cancer [81] and infectious diseases [82]. Immunogenicities of Gp96 [83], Hsp90 [84], Hsp70 [80,85], and Grp170/Orp150 [86] have been examined.

Cytotoxic immune cells including NK cells, CTL, and natural killer T (NKT) cells can recognize HSP and HSP peptides expressed on the membrane surface of tumor cells and infected erythrocytes by the receptor CD94, also known as killer cell lectin-like receptor D1 (KLRD1) [10,11], stimulating cytotoxicity with induced release of granzyme B [10,97]. Anticancer drugs caused the release of exosomes with HSPs from human hepatocellular carcinoma cells that elicited effective NK cell antitumor responses in vitro, suggesting that exosome membrane surface HSP may stimulate NK cells [73].

In the 1990s–2000s, clinical trials of HSP-based vaccinations immunomodulation were carried out, including autologous tumor-derived HSP peptide complexes (HSPPCs) [34,87,88], autologous tumor-derived Gp96 peptide complexes (HSPPC-96, Vitespen^®^, Oncophage), and recombinant oncolytic adenovirus overexpressing HSP70 (H103) [81] (Table 3). Intratumoral vaccination with a recombinant oncolytic adenovirus overexpressing the HSP70 protein eradicated primary tumors as well as inhibited the growth of established metastatic tumors in mice [81]. There were more than 150 medical centers worldwide in phase I/II/III clinical trials for many cancers. However, the limitations were also apparent, and specific alternatives have been developed.

DCs transfected with HSP70 mRNA (HSP70-DC) were tested in hepatitis C virus (HCV)-related hepatocellular carcinoma (HCC) as a phase I dose-escalation clinical trial in 2015 [98]. No adverse effects in grade III/IV were seen except one grade III liver abscess. Complete response (CR) without any recurrence occurred in two patients, stable disease in five patients, and progression of disease in five patients. Infiltration of CD8+ T cells and granzyme B in tumors was seen in immunohistochemistry.

ER chaperones such as binding BiP/HspA5 and Gp96/Grp94/TRA/Hsp90B1 play a multitude of roles within the ER; however, many of these chaperones can translocate to the cytosol and subsequently to the cell surface during ER stress [33]. On the cell surface or in the extracellular space, ER chaperones can play immunostimulatory roles in cancer, appearing as DAMPs recognized by innate and adaptive immune cells in the immune surveillance system in the host. Notably, BiP/HspA5 was found in HNSCC cells-derived EVs, although it was decreased in the high metastatic EVs compared to the low metastatic ones [1]. It was recently shown that phenotypically, distinct helper NK cells are required for Gp96-mediated anti-tumor immunity [99].

### 3.2. Anti-Inflammatory, Immunotolerant Roles of ex-HSP

It has been shown that immunization with HSPs has protective effects in induced arthritis models [100]. Immune reactivity to HSP has been found to result from inflammation in various disease models and human chronic inflammatory conditions, such as RA, type 1 diabetes, multiple sclerosis, and atherosclerosis [101]. Incubation with microbial HSP70 induced tolerogenic DCs and promoted an immunosuppressive phenotype in MDSCs and monocytes [102]. It was shown that Tregs (anti-inflammatory, immunosuppressive T cells) could recognize HSP70 self-antigens and enable Treg selective targeting to inflamed tissues [103]. HSPs are therefore attractive candidates for therapeutics in chronic inflammatory and autoimmune diseases, inducing long-lasting immune tolerance [77,104].

## 4. Receptors for ex-HSP and HSP Peptide Complex

Cell surface receptors known to be bound with ex-HSP90 are (i) CD91/LRP1/A2MR expressed on tumor cells, immune cells, and EVs, (ii) toll-like receptors (TLRs), (iii) scavenger receptor expressed by endothelial cells-1 (SREC-1), and (iv) CD94/KLRD1 expressed on killer cells. These receptors can be involved in the activation of intracellular signaling pathways, endocytosis, and immune response. The receptors expressed on the surface of EVs may hold ex-HSP90, as speculated in Figure 1.

### 4.1. CD91/LRP1/A2MR

CD91/LRP1/A2MR was shown to bind with HSP70, HSP90, and GP96 in macrophages [105]. Later studies identified CD91 as a key receptor of ex-HSP90 in cancer cells and skin cells [106]. ex-HSP90 binds to the subdomain II of CD91, and the intracellular NPVY motif (asparagine-proline-valine-tyrosine motif) is essential for activation of Akt1/2 signaling [27]. CD91 is expressed in hypoxic stress, plays a key role in endocytosis and transcytosis [107], and is thereby crucial in endocytosis of EVs and ex-Hsp90 in a hypoxic microenvironment. It was recently re-demonstrated that the establishment of tumor-associated immunity requires the interaction of HSPs with CD91 [108]. However, multiple receptors for ex-HSPs have been reported (Table 4).

### 4.2. Toll-Like Receptors (TLRs)

**Figure 2:**
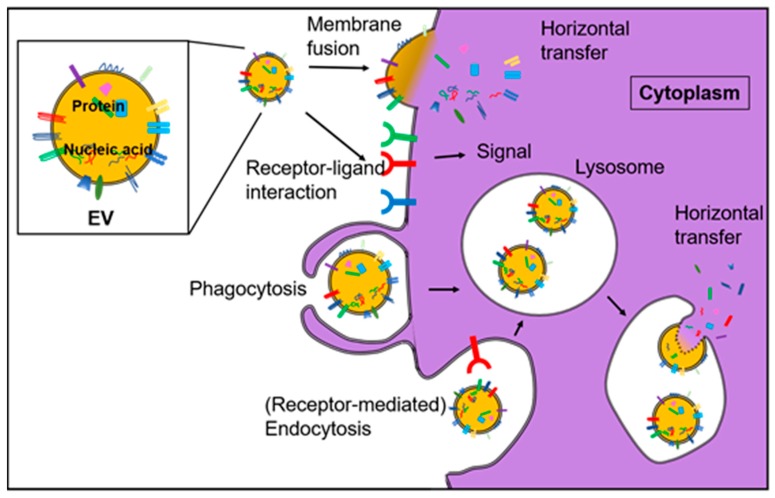
The multiple actions of EVs on/to the cells. The actions of EVs on cells are classified to (i) horizontal transfer of EV cargos dependently on membrane fusion, (ii) EV-surface ligand can activate cell surface receptors and subsequent signal transduction in the recipient cells, and (iii) macropinocytosis, phagocytosis or endocytosis. After the uptake, EV cargos can be (iv) functional, (v) recycled in recycling endosomes or (vi) processed in lysosomes.

It was first shown that mitochondrial molecular chaperone Hsp60 was a putative endogenous ligand of the TLR4 complex [109]. However, Hsp60 binding to macrophages occurred in the absence of surface TLR4, although no cytokine response was induced by Hsp60 in TLR4-deficient macrophages [105]. In addition, endogenous Hsp70 activated the Toll/IL-1 receptor signal pathway similar to Hsp60 and pathogen-associated molecular patterns (PAMP) [110]. In this study, Hsp70 induced IL-12 and activated a promoter of endothelial cell-leukocyte adhesion molecule-1 (ELAM-1, also known as E-selection or CD62E) in macrophages, while MyD88-deficient DCs did not respond to HSP70 with proinflammatory cytokine production. In the same journal, it was also reported that proinflammatory cytokine production induced by HSP70 was mediated by the MyD88/IRAK/NF-κB signal transduction pathway, for which HSP70 could use TLR2 (for gram-positive bacteria) and TLR4 (for gram-negative bacteria) to transduce proinflammatory signal CD14-dependently [111]. These studies indicated that (i) CD91/LRP1/A2MR can be a receptor of ex-HSPs and (ii) TLR2/4 can be receptors of ex-HSPs as well. In the latter case, any pathogen such as lipopolysaccharide (LPS), any other PAMP, or DAMP can be possibly contaminated within recombinant HSP fractions purified from bacteria inasmuch as diminishing contamination of pathogens may be difficult methodologically and technically. It has also been suggested that HSPs augment the ability of associated innate ligands such as LPS to stimulate cytokine production and DCs maturation [112]. Nevertheless, co-factors, co-receptors, co-stimulatory factors, or adaptor proteins might be of interest on the cell surface.

ex-HSPs/mHSPs on the surface of EVs can be recognized by receptors expressed on recipient cells, although multiple pathways have been suggested (Figure 2).

### 4.3. SREC-1

Scavenger receptors (SRs) are cell surface receptors for degenerated lipoproteins involving cholesterol and lipoprotein metabolism. SRs were first found in macrophages, whose SRs were used for internalization of denatured lipoproteins into cells. Interestingly, HSP70.PC vaccine activation of T cells required both TLR signaling and SREC-1 [113,114]. HSP70 peptide complex isolated from tumor-DC fusions (HSP70.PC-F) induced potent antitumor immunity. In this study, HSP70.PC-F-induced antitumor immunity depended on intact TLR signaling in immunized animals, while *Tlr2/Tlr4*-silenced mice did not respond to the HSP70 vaccine. Notably, TLR-dependent tumor cell-killing was inhibited by SREC-1 knockdown in DCs, suggesting a crucial role of SREC-1 in HSP70-mediated tumor immunity [113,115].

SREC-1 also plays a significant role in HSP90-mediated antigen cross-presentation [116]. HSP90- ovalbumin (OVA) peptide complexes bound to SREC-1 expressed on APCs. SREC-1 mediated internalization of HSP90-OVA polypeptide complexes via a Cdc42-regulated dynamin-independent endocytic pathway from early endosomes to recycling endosomes. It was shown that SREC-1 plays a primary role in HSP90-peptide complexes’ antigen uptake via cross-priming of MHC class I molecules as well as entry into the class II pathway [117].

In spite of ex-HSP, SREC-1 modulates the function of TLRs with essential roles in innate immunity. It has been shown that SREC-1 promoted dsRNA-dependent activation of TLR3 in monocytes [118]. SREC-1 also involved TLR4 entry into lipid microdomains and triggered the release of inflammatory cytokine in macrophages [119]. It was also shown that SREC-1 promoted dsRNA/CpG DNA-mediated TLR3/TLR9 activation followed by activation of NF-κB, interferon regulatory factor 3 (IRF3), and mitogen-activated protein kinase (MAPK) pathways, which trans-activate cytokine genes [120]. Additional SRs can bind with ex-HSP70, including lectin-like oxidized LDL receptor-1 (LOX-1), a member of both the c-type lectin receptor family and the SR family, as well as FEEL-1/CLEVER-1/STABILIN-1, containing multiple epidermal growth factor (EGF)-like repeats [121].

### 4.4. CD94, Killer Cell Receptor

CD94, also known as killer cell lectin-like receptor D1 (KLRD1), is a C-type lectin expressed on the surface of NK cells, CTL, and natural killer T (NKT) cells [10]. The CD94+ killer cells can recognize HSP70 and HSP70 peptides on the membrane surface of cancer cells and Plasmodium falciparum-infected erythrocytes [11], stimulating cytotoxicity with the induced release of granzyme B [10,97]. For activation of CD94+ killer cells, evident HSP-ligands are full-length ex-HSP70 [10], Hsp70 C-terminal domain, TKD (a 14-mer peptide derived from the N-terminal sequence of Hsp70 TKDNNLLGRFELSG, aa 450–463) [11,97,122,123], mHSP70 expressed on infected red blood cells [11], and tumor-derived HSP-exosomes [73]. IL-15/TKD treatment increased the proportion of CD94+ CD3+CD56+ NKT cells [122].

## 5. Inducible Mechanisms for HSPs

Heat shock factor 1 (HSF1) is a canonical transcription factor that mediates cell stress and induces the production of HSPs, although several additional transcription factors recently identified can be involved in cancer progression and resistance [3]. For expression of *HSP* genes and other target genes, HSF1 trimers bind to the heat shock elements (HSE) often located in promoter regions of these genes. It has been shown that PI3K-PKCδ signal mediates the activation of HSF1 and HIF-1, which co-trans-activate HSP genes [124], whose promoter regions are often enriched in the HSE and the hypoxia-responsive elements (HRE). Many types of HSP are induced upon hypoxia, including HSP70 [125], Orp150/Grp170 [126], Hsp27 [127], and HSP90 [9,128], along with the induction of vascular endothelial growth factor (VEGF) [129] and heme oxygenase-1 (HO-1) [130].

The hypoxic environment in tumors is essential for the production of ex-HSP. Tumor hypoxia is a distinguishing feature of solid tumors resulting from the inadequate oxygen delivery of the abnormal blood vessels supplying the tumor that cannot meet the demands of the rapidly proliferating cancer cells [131,132,133]. For example, molecular targeting of C-X-C motif chemokine receptor 4 (CXCR4) on vascular endothelial cells induced tumor angiogenic inhibition triggered necrosis (TAITN) in oral cancer, although HIF-1α was induced in the hypoxic and the necrotic tumor tissue [134]. Intratumoral hypoxic stress induces HIF-1 that trans-activates a number of target genes, including *HSP90AA1* gene encoding HSP90α [5,9,135], ATP-binding cassette (ABC) transporter genes such as *ABCG1* and *ABCG2* [74], *MMP* genes, and connective tissue growth factor (*CTGF)/CCN2* gene [136,137].

Secreted ex-HSP90α and ex-HSP90β were found in the conditioned media of breast cancer cell lines, in which HIF-1α is constitutively active [26]. In breast cancer MDA-MB-231 cells, the secreted ex-HSP90 increased cancer cell survival in a hostile hypoxic environment via CD91-mediated activation of Akt, a kinase mediating cell survival. The three-dimensional (3D) tumor organoid (tumoroid) culture system enabled reproduction of intratumor hypoxia with CSC properties from which ex-HSP90 was abundantly released, although not from two-dimensional (2D)-cultured normoxic cells [5,138].

HIF-1 signaling stimulates the migration of human dermal fibroblast (HDF) by inducing the HSP90α secretion into the extracellular environment [9,139]. The secreted ex-HSP90α, in turn, promotes the hypoxia cell motility signaling. Interestingly, recombinant HSP90α treatment greatly doubled the hypoxia effect on HDFs. On the other hand, antibody blockade of ex-HSP90α completely abrogates the hypoxia–HIF-1 pathway-stimulated HDF migration [106,140]. Transforming growth factor-alpha (TGFα), a member of the EGF ligand family, also stimulates the secretion of HSP90α [106]. CD91/LRP1 has been identified as a key receptor of ex-HSP90 [106]. It is thought that EV-HSP90 could also bind to and stimulate LRP1 on the surface of recipient cells (Figure 2). Therefore, tumor hypoxia induces LRP1 and HSP90 expression and LRP1-HSP90 interaction on the surfaces of cells, and EVs could promote tumor growth.

HSP90 co-works with more than 10 types of co-chaperones [63]. Cell division control 37 (CDC37) is a kinome co-chaperone of HSP90 [62,70]. Notably, two SREZBP-CTfin51-AW1-Number 18 cDNA (SCAN)-type transcription factors—myeloid zinc-finger 1 (MZF1) and SCAN domain-containing protein 1 (SCAN-D1)—were shown to reciprocally regulate cell division control 37 (CDC37); the former activates and the later represses the *CDC37* gene [62,70]. These transcription factors regulate genes encoding molecular chaperones and co-chaperones and are thus crucial in cancer progression and resistance.

Recent studies have discovered that intracellular matrix metalloproteinase-3 (MMP-3) and heterochromatin protein 1 (HP1), also known as chromobox proteins (CBX), activate *HSP72, HSP70B’,* and *HSP27* [68].

## 6. HSPs as Biomarkers Detectable by Liquid Biopsies

**Figure 3:**
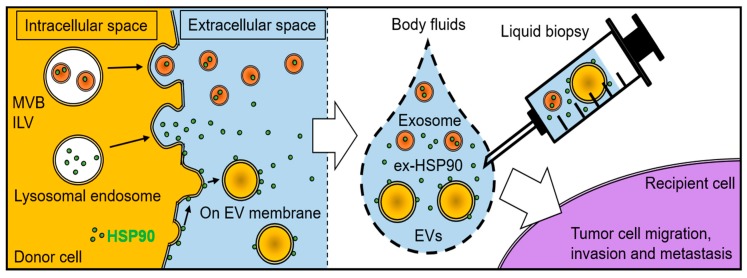
Liquid biopsy for diagnosis, prognosis, and treatment of diseases. Liquid biopsy is applicable to blood, saliva, urine, stool, semen, sweat, tear, nasal mucus, milk, and cerebrospinal fluid. Blood analytes are composed of cell-free proteins, circulating cell-free DNA (cfDNA), circulating cell-free RNA (cfRNA), circulating cell-free microRNA (cfmiRNA), circulating tumor DNA (ctDNA), EVs e.g. exosomes or oncosomes, circulating tumor cells (CTCs), tumor-educated blood platelets (TEPs), and metabolites. HSPs can be released from tissues upon cellular/tissue stress, damage, cell death, and hypoxia. Such ex-HSPs in body fluids are free proteins, protein complex, ribonucleoprotein (RNP) complexes, or HSP-rich EVs. ex-HSPs also belong to alarmins, DAMPs, or tumor associated antigens (TAAs) stimulating the host immune system. HSPs in body fluids may be a diagnostic or prognostic value as a cancer biomarker. The depletion of HSPs in the blood may prevent cancer progression and resistance.

HSPs are often released from tissues into body fluids upon cellular/tissue stress, damage, cell death, hypoxia in cancer progression, and other various pathological conditions, known as alarmins, DAMP or chaperokines (Figure 3). These released HSPs are cell-free proteins, protein complex, ribonucleoprotein (RNP) complex [2], membrane-associated HSPs, vesicle surface-associated HSPs, or cargos packaged in EVs such as exosomes and oncosomes [141].

### 6.1. Serum HSP Biomarkers

Hsp70 is often found on the plasma membrane of malignant tumors and released into the blood circulation with exosomes. It was suggested that an mHSP70 phenotype correlates with tumor aggressiveness, whereas such mHsp70s are targeted by cytolytic immune cells such as NK cells. It was indeed shown that squamous cell non-small-cell lung cancer (NSCLC) and adeno NSCLC patients harbored significantly higher levels of serum Hsp70 than healthy controls [15]. Serum Hsp70 levels were significantly correlated with the gross tumor volume in both types of NSCLC. This study also indicated that NK cells can be more active in squamous cell carcinoma (SCC) than adenocarcinoma, suggesting different levels of NK immunity. Even in pet dogs, serum Hsp70 levels were demonstrated as a potential diagnostic biomarker for spontaneous round cell tumors [142]. Free HSPs and exosome HSPs can be attractive biomarkers for diagnosis and prognosis in cancer, including HNSCC [1] and prostate cancer [5] as well. EVs secreted by high-metastatic HNSCC cells contained high amounts of TRAP1, Hsp90β, Hsp90α, Hsp105/HspH1, and Hsp72 compared to low-metastatic HNSCC cells [1]. Indeed, patients harboring TRAP1-high or HSP90β-high tumors are correlated with poor prognosis compared to low-expression patients’ groups. In HNSCC patient cases, high expression of TRAP1 and Hsp105 was found over the stages (I to IV), while HSP90α/β-high expression cases were increased in later stages (stages II to IV) compared to stage I cases [1]. Besides, ex-HSP90α was abundantly secreted by enlarged 3D hypoxic tumoroids formed with castration-resistant prostate cancer (CRPC) cell line PC-3, although not by smaller tumoroids nor by 2D-cultured cells [5]. In this model, ex-HSP90α was abundantly released, while EV-HSP90α was barely detected.

Besides, serum Hsp70 has been shown to be increased after exercise and acute terms through induction by IFN-γ and elevated body temperature, i.e., thermal stress [16]. For example, after ironman triathlon races, Hsp70 was released into the blood circulation as a function of exercise duration, indicating that Hsp70 is released into circulation upon tissue damages, while anti-inflammatory cytokines were induced, and pro-inflammatory cytokine response was minimal [143]. Recent studies indicated that systemic ex-Hsp72 in plasma is associated with a potential diagnostic or predictive biomarker of sarcopenia [17]. Consistently, it has been shown that sarcopenia is associated with increased systemic inflammation in older adults.

Extracellular small HSPs have been demonstrated to be involved in various pathological conditions. The most frequently studied member of a small HSP family is Hsp27/HspB1, which is overexpressed in various cancers and is associated with tumor metastasis, progression, and a poor prognosis. Elevated serum levels of HSP27 have been detected in patients with breast [144], ovarian [145], and colon cancer [146], hepatocellular carcinoma [147], gastric adenocarcinoma [148], as well as chronic pancreatitis [149], diabetic neuropathy [150], and insulin resistance [151]. In addition, serum HSP27 has been detected in patients with atherosclerosis [152,153], acute coronary syndrome, and reperfusion after ischemic clamping during heart bypass surgery [154]. HSP27 has also been found in the cerebrospinal fluid during spinal cord and brain ischemia [155].

### 6.2. Serum Antibodies against HSP (60, 70, and 90)

Besides, HSPs belong to TAAs overexpressed in various human cancers. Elevated HSP can stimulate the immune system to produce anti-HSP autoantibodies (AAbs). AAbs against HSPs have been identified in the circulation of various cancer patients [156]. Because of their specificity and stability in the sera, AAbs against HSPs can be also attractive biomarkers for the development of less invasive serological tests for the diagnosis and the prognosis of cancer.

### 6.3. Cancer Liquid Biopsies and HSPs

Tissue biopsies are commonly used for diagnostic, prognostic, and treatment purposes, despite a lot of limitations such as its invasive nature, failure to reflect the tumor heterogeneity, and the discomfort suffered by the patient. Liquid biopsies are considered as a non-invasive alternative to tissue biopsies and can provide advanced diagnostic information from a small amount of body fluid such as blood. Notably, liquid biopsy has attracted attention as an important technique for early detection and diagnosis of cancer and various pathological conditions [157] by using blood [158,159], saliva [160,161,162], urine [163,164,165], stool [166,167], semen, sweat, tear, nasal mucus, breast milk, cerebrospinal fluid, amniotic fluid, and malignant ascites [168,169,170,171] (Figure 3).

Liquid biopsy can derive genetic, epigenetic, and proteomic information of multiple cancers at one time, estimate the cellular and the molecular characteristics of cancer-associated cells, and monitor the response to different anticancer therapies [172]. Blood analytes are composed of circulating cell-free DNA (cfDNA), including mutant DNA and methyl DNA, circulating cell-free RNA (cfRNA) including mRNA and circulating cell-free microRNA (cfmiRNA), cell-free proteins, circulating tumor DNA (ctDNA), EVs such as exosomes, circulating tumor cells (CTCs), tumor-educated blood platelets (TEPs), and metabolites [172,173,174,175,176,177]. Liquid biopsies of CTCs, circulating cell-free materials, and EVs are useful for early detection of cancer and prognostic assessment of cancer progression dynamics [157,178].

Thus, ex-HSP and exosome-HSPs in body fluids may be useful for theranostics of cancers in combination with other biomarkers detectable by liquid biopsies.

## 7. HSP-Targeted Therapies

Targeting HSPs at protein and mRNA levels has been tried inasmuch as HSPs are anti-apoptotic resistant chaperones in tumor cells, although extracellular and vesicular HSPs are under investigations. In this section, we review HSP-targeted inhibitors, HSP mRNA-targeted therapeutics, clinical trials, and potential nano-vesicles delivering drugs.

### 7.1. Clinical Trials of HSP90 Inhibitors

The first discovered HSP90 inhibitor was geldanamycin (GA), belonging to the benzoquinone ansamycin antibiotics [179]. GA was found to arrest the tumor proliferation by inhibiting the Src tyrosine kinase activity, although it was unable to directly inhibit the activity of purified Src kinase [180,181]. Further studies revealed that the anti-proliferative effect of GA resulted from its binding to the ATP binding pocket of HSP90. Consequently, GA inhibited the binding of the client proteins to HSP90 and led to the proteasomal degradation of these proteins. These results proved that the efficacy of HSP90 inhibitors is closely related to their binding ability with HSP90.

In order to reduce the hepatotoxicity and increase water solubility, the structure of GA was modified to generate 17-allylamino-17-demethoxygeldanamycin (17-AAG), also known as tanespomycin. The 17-AAG was the first HSP90 inhibitor used in human clinical trials [182] (Table 5). Although the 17-AAG is still insoluble in water, a considerable effect was observed in clinical phase I trials. In addition, the phase II trials were performed on patients with metastatic breast cancer and melanoma, and side effects such as tiredness, nausea, diarrhea, and liver damage were reported, by which the use of 17-AAG was stopped [183].

Several HSP90 inhibitors, including Ganetespib, AUY922, and Retaspimycin, were tested in phase III clinical trials in non-small cell lung cancer (NSCLC), although none were positive in unselected NSCLC [192]. Therefore, drug development was halted. However, results were more promising in anaplastic lymphoma kinase (ALK)-rearranged NSCLC patients. Overexpression of Hsp27 in squamous NSCLC is a mechanism of chemoresistance. Therefore, Hsp27 inhibitor Apatorsen was tested in squamous NSCLC.

A number of HSP90 inhibitors inhibit ATP hydrolyzing activity by binding to the ATP-binding site of HSP90 and suppress its chaperone function required for client proteins conformation changes. Such an effect of HSP90 inhibitors decreases the binding affinity of the client proteins to the HSP90, resulting in their dissociation from HSP90. The client proteins become structurally unstable, ubiquitinated, and degrade by the proteasome. The reduction of client oncoproteins prevents the growth of cancer cells. The most surprising finding with HSP90 inhibitors is their higher affinity and selectivity towards the tumor cells and not to the normal cells [193].

To overcome this drawback, combining the HSP90 inhibitors with other drugs [194,195] and/or radiation [196,197] has been investigated, although these are still under investigation. Most recently, an HSP90 inhibitor XL888 in combination with a v-Raf murine sarcoma viral oncogene homolog B1 (BRAF) inhibitor Vemurafenib had clinical activity in patients with advanced BRAF-V600-mutant melanoma with a tolerable side-effect profile [198], while it was indicated that HSP90 inhibitors warrant further evaluation in combination with current standard-of-care (SOC) BRAF plus MEK inhibitors in BRAF-V600-mutant melanoma (Table 5).

Kang et al synthesized and biologically evaluated a novel 18F-labeled 5-resorcinolic triazolone derivative (1, [18F] PTP-Ganetespib) based on Ganetespib, a most promising candidate among several HSP90 inhibitors under clinical trials, which entered “phase III” clinical trials for cancer therapy [199]. [18F] PTP-Ganetespib was highly taken up in breast cancer cells, including triple-negative breast cancer (TNBC) MDA-MB-231 and Her2-negative MCF-7 cells. [18F] PTP-Ganetespib was retained longer in the tumor than other organs, shown in biodistribution and microPET imaging studies.

### 7.2. Potential Limitations of HSP90 Inhibitors

ATP-independent activities of HSP90—although most HSP90 inhibitors target the ATP binding site, chaperokine activities of ex-HSP90 and EV-HSP90 are not dependent on the ATP hydrolyzing activity. EV-HSP90 incorporated within the EVs could be propagated in the tumor microenvironment and in body fluids and is not easily targeted by the small molecule chemical inhibitors. EV-mediated RASP could promote the release of HSP90 inhibitors with EVs.

The physiological necessity of HSP90 and target cell selectivity—HSP90 is required for homeostasis of normal, non-cancerous cells. Without a cancer cell-targeted drug delivery system (DDS), HSP90 inhibitors could be harmful and toxic to normal cells, leading to unfavorable side effects (potential usefulness of nano-vesicles such as DDS is mentioned below.) Notably, HSP90β is a housekeeping protein whose activities are essential in all cells. Besides, HSP90α is an inducible protein essential for physiological stress response in normal cells.

HSP90/HSF1 feedback system—HSP90 binds to and keeps the inactivated status of HSF1, whereas HSP90 inhibitors trigger the release of HSF1 from the HSP90/HSF1 complex and the subsequent trans-activation of HSP genes, e.g., a compensatory increase in HSP70 expression and other numerous genes, which induce a stress response and resistance of cancer cells. HSF1 is a stress-responsive transcription factor and has been reported as a multi-faceted modulator of tumorigenesis [66,200,201,202,203,204]. In response to heat shock stress [68,202,205,206], intracellular accumulation of misfolded proteins [3,207,208,209,210,211,212], or tumor-promoting signaling such as phosphatidylinositol 3-kinases (PI3K)-Akt-mTOR signaling [200,213], HSF1 is activated and translocated into the nucleus, where it binds to HSP genes promoters and fosters their transcription. HSF1 transcriptional activity can be regulated through feedback inhibition by HSP90 [38,214,215,216]. Therefore, HSP90 inhibitors could trigger the release of HSF1 from the HSP90/HSF1 complex and de-repress HSF1, which is then able to trans-activate a number of HSP genes and oncogenes [214,215]. Importantly, these stress-responsive genes and the up-regulation of oncogenes enable tumor cells to respond to a variety of stresses and allow them to thrive in unfavorable growth conditions. Thus, the HSP90/HSF1 feedback system could counteract the cell-killing (cytotoxic) effect of HSP90 inhibitors.

### 7.3. HSP70 Inhibitors

The human HSP70 family contains several highly homologous members composed of N-terminal nucleotide-binding domain (NBD) containing ATPase activity, substrate-binding domain (SBD), and C-terminal EEVD domain binding to co-chaperone DnaJ/HSP40, nucleotide exchange factors (NEF), and B-cell lymphoma 2 (BCL2)–associated anthanogene 3 (Bag3) [2]. Inhibitors binding each domain have been developed and tested. The HSP70 SBD is targeted by ADD70 polypeptide, 2-phenylethynesulfonamide (PES/Pifithrin μ), PES-CI, and A8 peptide. The HSP70 NBD is targeted by a number of inhibitors, including VER155008, MKT-077, and YK5. The HSP70 EEVD domain is targeted by YM-1, Myricetin, MAL3-101, and 15-deoxyspergualin [217,218]. The 15-deoxyspergualin was tested in a phase II clinical trial in metastatic breast cancer, although neuromuscular side effects with no benefit for the disease were reported [219]. See our recent review of the HSP70 family and inhibitors for more information [2].

### 7.4. Anti-mHSP70 Antibody

Antibody based-therapy had been considered the best strategy for targeting HSP70, although it was hindered by the lack of specificity to tumor markers [220]. However, Stangl et al. developed a monoclonal antibody mHsp70.1 specifically recognizing the extracellular 14-mer motif TKDNNLLGRFELSG (TKD) of membrane-bound HSP70 (mHSP70). It is worth noting that the membrane form of Hsp70 is frequently expressed in tumors but not on normal cells [221]. The safety of CmHsp70.1 has been proven, and it passed the phase I clinical trial and the phase II clinical trial for NSCLC in combination with radiochemotherapy in 2005 [222].

### 7.5. HSF1 Inhibitors

The bioflavonoid quercetin was the first approach to inhibit transcriptional factor HSF1, thereby inhibiting the HSP70 induction at the mRNA levels [223]. Subsequently, more potent compounds inhibiting HSF1 are a benzylidene lactam compound, KNK437 [224], and a triazole nucleoside analog [225]. However, targeting HSF1 blocks the transcription of all stress-inducible HSPs and other transcriptionally targeted genes, thus disturbing the housekeeping functions of these proteins in normal cells and various pathophysiological conditions [226].

### 7.6. HSP40 Inhibitors

HSP40 is a co-chaperone of HSP70, stimulating HSP70 ATPase activity and thereby compromising HSP70 functions. A series of phenoxy-N-arylacetamides was identified to directly bind to bacterial Hsp40/DnaJ and disrupted Hsp70/Hsp40-mediated luciferase refolding by binding to DnaJ [227].

Chalcone C86 was recently identified to interact with Hsp40, thereby destabilizing androgen receptor variants-mediated transcriptional activities in CRPC [228].

### 7.7. HSP110 Inhibitor

HSP110, an HSP70 nucleotide-exchange factor, is necessary for cancer cell survival. A mutant of HSP110 (HSP110DeltaE9) not only diminished HSP70 chaperone activity but also sensitized cancer cells to anticancer drugs both in vitro and in vivo [229].

### 7.8. HSP27 Inhibitors

HSP27/HspB1 is a potent cytoprotective chaperone, and it is overexpressed in many cancer types; thus, it is an attractive target in cancer therapy. However, HSP27-directed therapy is difficult, as HSP27 is an ATP-independent chaperone, unlike other HSPs [230]. There are three approaches that have been reported for HSP27 inhibition: (i) by small molecules inhibitors that directly bind to HSP27 and inhibit its downstream pathways. These compounds include RP101 (Brivudine) [231] and Zerumbone [232]. Zerumbone was directly inserted between the disulfide bonds in the HSP27 dimer and modified normal HSP27 dimerization; (ii) using peptide aptamers, which specifically bind to HSP27 and disturb the dimerization and the oligomerization of the chaperone [233]; (iii) OGX-427 antisense oligonucleotides that target HSP27 mRNA [234]. It is worth noting that both small molecule inhibitors of HSP27 and peptide aptamers are used to enhance chemotherapy efficacy of anti-cancer drugs, as they are not effective on their own. On the other hand, OGX-427 is the only HSP27 inhibitor that successfully passed through phase I clinical trials and is currently being tested in phase II/III [188,235].

### 7.9. HSP mRNA-Targeted Therapy

The above-mentioned OGX-427 is most promising as *HSP27* mRNA-targeted therapy. Besides, HSP70 siRNA was used for “blocking heat shock response” in MnO2/Cu2-xS-based multimode imaging diagnostic and advanced single-laser irradiated photothermal/photodynamic therapy [236]. In this study, Cao et al. used MnO2 to relieve tumor hypoxia inasmuch as MnO2 was reduced to Mn2+ ion and triggered the decomposition of H2O2 into O2 in the tumor acidic microenvironment.

HSP90-rich EVs are released by metastatic cancer cells, whereas small interfering RNA (siRNA) double-targeting HSP90α and HSP90β mRNAs efficiently decrease cancer cell viability, indicating a novel concept of HSP90 mRNA-targeted oligonucleotide therapeutics [1]. Most HSP90 inhibitors target ATP-binding pockets, although the ATP-dependent activity of HSP90 in cells and extracellular space has been assumed.

### 7.10. Nano-Vesicles as Potential DDS

Nanovesicles such as exosomes and liposomes were recently examined as DDS delivering drugs. Nanovesicles delivering HSP90 inhibitors or HSP mRNA-targeted RNAi may be effective in killing cancer cells or inhibiting tumor heat shock responses. Specific vesicle-surface molecules, e.g., cancer-cell targeting ligands, “do not eat me” signals, and masking “eat me” signals, may be required for selective targeting on cancer cells and avoid phagocytosis by macrophages.

Nevertheless, the tumor microenvironment can be also targeted, e.g., CXCR4+ TEC was targeted by AMD3100 to induce tumor angiogenic inhibition triggered necrosis (TAITN) [134]. Therefore, HSP in TEC, CAF, tumor-associated macrophages (TAM), and immunosuppressive cells could be targeted in the future.

## 8. Conclusions

Extracellular HSPs including HSP90 (α, β, Gp96, Trap1), HSP70, and large and small HSPs have been found in exosomes, oncosomes, membrane surfaces, as well as free HSP in cancer and various pathological conditions, also known as alarmins. Oncosomes released by tumor cells are a major aspect of RASP by which immune evasion can be established. Releases of ex-HSP and HSP-rich oncosomes are essential in RASP, promoting cancer progression and resistance against host immunity and therapeutic stress. RASP of tumor cells can eject anticancer drugs. Cytotoxic lipids can be also released from tumor cells as RASP. ex-HSP and mHSP play immunogenic roles recognized by APCs, leading to T cell cross-priming, as well as by CD94+ NK cells, leading to tumor cytolysis, whereas ex-HSP/CD91 signaling in cancer cells promotes cancer progression. HSP genes are canonically activated by HSF1 and hypoxia signaling mediated by HIF-1, while matrix metalloproteinase (MMP)-3 and HP1 are novel positive regulators for inducible types of HSPs. HSPs in body fluids are potential biomarkers analytical by liquid biopsies in cancers and tissue-damaged diseases. Some HSP-based vaccines, inhibitors, and RNAi therapeutics are promising in cancer therapy.

## Figures and Tables

**Table 1:** HSPs Found Extracellularly, in Exosomes and on Membranes.

Subfamily Name	Prototypical Members	Exosome HSP	Free HSP	Membrane HSP
**HSP90**	Hsp90α ^HSP90AA1^ Hsp90β ^HSP90AB1^ Gp96 ^Grp94/Hsp90B^ TRAP1 ^Hsp75/Hsp90L^	Cancer [1] B cell [6]	3D Tumoroid [5] HDF [9]	Tumor cells APCs Infected erythrocyte [10,11,12]
**HSP70** **HspA(1-12)**	Hsp72 ^HspA1A/HspA1B^ Hsc70, BiP ^HspA5^ Hsp70B’ ^HspA6^ See [2] in detail	Cancer [1,13] B cell [6]	Mφ [14], Cancer [5,15] Exercise [16] Sarcopenia [17]	Cancer [18,19]
**Small HSP** **HspB(1–10)**	Hsp27 ^HspB1^ αB-crystallin ^HspB5^	B cell [6], Sera, body fluids [8]	Sera, body fluids [8]	?
**Large HSP**	Hsp105, Hsp110 Grp170 ^Orp150^	Cancer [1]	?	?
**HSP40** **DnaJ**	Tid1^DnaJA3^ [20] ERdj4 ^DnaJB9^ RME8 ^DnaJC13^	?	?	?
**HSP47**	Colligin-2 ^RA-A47^	?	RA sera [21]	Chondrocyte [22]
**Chaperonin**	TRiC ^CCT^ Hsp60, Hsp10	Cancer [1,23]	DM [24]	?

Cell damages in pathological conditions induce expression and release of inducible types of HSPs, often found in extracellular space as free forms, on the membrane surface, and in vesicles, although constitutively expressed types of HSP is also found extracellularly. ?, unknown; RA, rheumatoid arthritis; DM, diabetes mellitus; HDF, human dermal fibroblast; APC, antigen-presenting cell.

**Table 2:** Immunomodulatory Roles of Extracellular HSP as Vaccines.

	Immunostimulatory HSP	Immunotolerant HSP
Target diseases	Cancers Infectious diseases	Rheumatoid arthritis Type 1 diabetes Atherosclerosis Multiple sclerosis
APC	DCs Mφ	Tolerogenic DCs
Immune cells	Antigen-specific CD8+ CTL NK cells, NKT cells	MDSC Treg
HSP antigens	Gp96 ^Grp94/TRA^, BiP ^HspA5^ HSP90, HSP70 Grp170 ^Orp150^ Small HSPs	Microbial HSP70/HSP60
Effects	Antigen cross-presentation T cell cross-priming Tumor cytolysis	Immune tolerance Anti-inflammatory Immunosuppressive

MDSC, myeloid-derived suppressor cell; NK, natural killer; NKT, natural killer T; DC, dendritic cell; Mφ, macrophage; CTL, cytotoxic T lymphocyte.

**Table 3:** HSP-Based Immunotherapy Trials.

Concept, Material	Disease	Phase	Note, Outcome
Autologous tumor-derived HSP peptide complexes (HSPPCs)	RCC Melanoma	III	Had clinical activities. In Phase III trials for advanced melanoma and RCC patients, efficacy, safety, and feasibility were demonstrated [34,87,88]. However, the limitations were apparent, and specific alternatives have been developed.
	CML, CRC LymphomaPancreatic cancer Gastric cancer	I/II	
Autologous tumor-derived HSP Gp96-peptide complexes HSPPC-96 Vitespen^®^ Oncophage	RCC Metastatic melanoma	III	Feasible, devoid of significant toxicity, induced clinical and tumor-specific T-cell responses in vaccinated patients [89,90]. Promising in enhancing survival of patients [91,92].
	CRC, RCC Glioblastoma Lung cancer Melanoma	I/II	Almost devoid of side effects aside from minor injection-site reactions [93].
Preparation of HSPPC-96	Pancreatic adenocarcinoma	I	No correlation between immune response and prognosis. Feasible prep of HSPPC-96 [94].
HSPPC-96 + GM-CSF + IFN-α	Metastatic melanoma	II	Gained tumor-specific T cell-mediated and NK responses, but immune, clinical responses were not gained compared with monotherapy [95].
Recombinant oncolytic adenovirus overexpressing HSP70 (H103)	Advanced solid tumors	I	CR + partial response was 11.1% (3/27), and the clinical benefit rate (CR + partial response + minor response + stable disease) was 48.1%. CD4+ and CD8+ T cells and NK cells were elevated [96].
Dendritic cells transfected with HSP70 mRNA (HSP70-DC)	HCV-related HCC	I	Safe and feasible. Almost no adverse effects in grade III/IV. CR without any recurrence (2), stable disease (5), a progression of the disease (5). Infiltrating CD8+T cells and granzyme B in tumors.

RCC, renal cell carcinoma; CML, chronic myelogenous leukemia; CRC, colorectal cancer; GM-CSF, granulocyte macrophage colony-stimulating Factor; IFN, interferon; CR, complete response; HCV, hepatitis C virus; HCC, hepatocellular carcinoma.

**Table 4:** Receptors for ex-HSPs and HSP Peptide Complex.

Receptor	Key events	Expression	Notes
CD91/LRP1/A2MR	Hypoxia response EMT Antitumor immunity	Cancer cell APCDermal fibroblast Exosome	Mediating ex-HSP-triggered endocytosis. Mediating ex-HSP activation of MEK-ERK signaling and Akt signaling. Hypoxia stimulates production of ex-HSP and ex-HSP/CD91 axis. EMT can be stimulated by ex-HSP/CD91 axis. Interaction of tumor-derived HSP with CD91 on APC is the host-priming of T-cell antitumor activity.
TLR2 TLR3 TLR4 TLR9	Immune response DAMP/PAMP signal	APC Epithelial cell	Mediating ex-HSP DAMP signaling to stimulate innate immunity and cytokine production. CD14 and SREC-1 may be essential for the ex-HSP/TLR signaling.
SREC-1	Immune response Antigen cross-priming	APC	Crucial in APC-mediated immune response. Essential for ex-HSP/TLR signaling. Essential in HSP90-peptide complexes antigen uptake through cross-priming of MHC class I molecules and entry into the class II pathway.
CD94/KLRD1	Cytotoxicity targeting tumor and infected cells	NK cell CD8+ CTL NKT cell	CD94+ NK, NKT, and CTL can recognize Hsp70 and Hsp70 peptides on cancer cells and infected RBC, stimulating cytotoxicity. Evident ligands are (i) full-length HSP70, (ii) Hsp70 C-terminal domain, (iii) a 14-mer peptide of Hsp70 N-terminus, TKDNNLLGRFELSG, named TKD, (iv) membrane-surface HSP70 expressed on infected RBC, (v) tumor-derived HSP-exosomes.

EMT, epithelial to mesenchymal transition; DAMP, damage-associated molecular patterns; PAMP, pathogen-associated molecular pattern; TLR, Toll-like receptor; KLRD1, killer cell lectin-like receptor D1; SREC, scavenger receptor expressed by endothelial cells-1; MHC, major histocompatibility complex; RBC, red blood cells.

**Table 5:** HSP-Based Trials.

Concept	Disease	Phase	Note, Outcome
HSP90 inhibitor 17-AAG	Metastatic breast cancer Melanoma	I/II	Side effects occurred such as tiredness, nausea, diarrhea, and liver damage. HSP70 was induced in PBMC [184,185].
HSP90 inhibitor Ganetespib^®^	NSCLC	III	Not positive in unselected NSCLC. Therefore, drug development was halted. More promising in ALK-rearranged NSCLC patients.
HSP90 inhibitor Retaspimycin^®^	NSCLC	III	
HSP90 inhibitor AUY922	NSCLC	III	
	Stage IV NSCLC	II	Active particularly among patients with ALK rearrangements and EGFR mutations [186].
HSP90 inhibitor AUY922 + Erlotinib	EGFR-mutant lung cancer	I/II	Evaluated in acquired resistance to EGFR-TKI. Partial responses, but the duration of treatment was limited by toxicities, especially night blindness. Did not meet its primary endpoint [187].
HSP90 inhibitors	CRPC	I/II	Negligible anticancer activity and dose-limiting toxicity profiles [188].
Oral HSP90 inhibitor PF-04929113 (SNX5422)	Recurrent, refractory hematologic malignancies	I	Alternate-day oral dosing at 74 mg/m (2) for 21/28 days was tolerated with reversible toxicity. Myeloma and lymphoma patients were responsive [189].
Oral HSP inhibitor Debio0932	NSCLC Breast cancer	I	Has limited clinical activity with manageable toxicity [190].
HSP27-targeted antisense oligonucleotide OGX-427 Apatorsen^®^	Squamous NSCLC	I	Tested, as overexpression of Hsp27 in squamous NSCLC is a mechanism of chemoresistance.
	Metastatic non-squamous NSCLC	II	A combination of carboplatin and pemetrexed was evaluated. Well tolerated but did not improve outcomes in the first-line setting [191].
	Advanced bladder cancer	II	A combination of cisplatin and apatorsen was tested.
	CRPC	II/III	Has shown good biological activity [188].
HSP70 inhibitor 15-deoxyspergualin	Metastatic breast cancer	II	Neuromuscular side effects with no benefit for disease.
Anti-HSP70 antibody recognizing TKD	NSCLC	I/II	Safe in phase I. Evaluated in combination with radio-, chemotherapy.

17-AAG, 17-allylamino-17-demethoxygeldanamycin; PBMC, peripheral blood mononuclear cells; NSCLC, non-small-cell lung cancer; ALK, anaplastic lymphoma kinase; EGFR, epidermal growth factor receptor; CRPC, castration-resistant prostate cancer.

## Abbreviations

17-AAG	17-allylamino-17-demethoxygeldanamycin
A2MR	Alpha 2 macroglobulin receptor
AAb	Autoantibody
ABC	ATP-binding cassette
APC	Antigen-presenting cell
BiP	Binding immunoglobulin protein
CAF	Cancer-associated fibroblast
CBX	Chromobox protein
CDC37	Cell division control 37
CDK	Cyclin-dependent kinase
CIC	Cancer-initiating cell
CML	Chronic myelogenous leukemia
CR	Complete response
CRC	Colorectal cancer
CRPC	Castration-resistant prostate cancer
CSC	Cancer stem cell
CTC	Circulating tumor cell
ctDNA	Circulating tumor DNA
CTGF	Connective tissue growth factor
CTL	Cytotoxic T-lymphocyte
CXC	Cysteine-X-cysteine motif
DAMP	Damage-associated molecular pattern, danger-associated molecular pattern
DM	Diabetes mellitus
EGFR	Epidermal growth factor receptor
EMT	Epithelial to mesenchymal transition
EV	Extracellular vesicle
EV-Hsp	Extracellular vesicle-associated heat shock protein
ex-Hsp	Extracellular HSP
FcR	Fragment-crystallizable receptor
GP96	Glycoprotein 96
GRP	Glucose-regulated protein
HIF	Hypoxia-inducible factor
HP1	Heterochromatin protein 1
HSF	Heat shock factor
HSP	Heat shock protein
ILV	Intra-luminal vesicle
IRAK	IL-1 receptor-associated kinase
LPS	Lipopolysaccharide
LRP1	Low-density lipoprotein receptor-related protein 1
MDSC	Myeloid-derived suppressor cells
MHC	Major histocompatibility complex
MMP	Matrix metalloproteinase
MSC	Mesenchymal stem cell
mTOR	Mammalian target of rapamycin
MV	Microvesicle
MVB	Multi-vesicular body
Myd88	Myeloid differentiation 88
MZF1	Myeloid zinc finger 1
NK	Natural killer
NSCLC	Non-small-cell lung cancer
OncomiR	Oncogenic microRNA
ORP150	Oxygen-regulated protein 150
OSCC	Oral squamous cell carcinoma
PAMP	Pathogen-associated molecular pattern
PD-1	Programmed cell death-1
PD-L1	Programmed cell death-ligand 1
PI3K	Phosphatidylinositol 3-kinases
POC	Proof of concept
RA	Rheumatoid arthritis
RASP	Resistance-associated secretory phenotype
RCC	Renal cell carcinoma
SCAN	SREZBP-CTfin51-AW1-Number 18 cDNA
SCC	Squamous cell carcinoma
SR	Scavenger receptor
SREC	Scavenger receptor expressed by endothelial cells-1
TAA	Tumor-associated antigen
TAITN	Tumor angiogenic inhibition triggered necrosis
TLR	Toll-like receptor
TRAP-1	TNF receptor-associated protein-1
Treg	Regulatory T cells
Tumoroid	Tumor organoid
